# Supplementary material for: Alternative Architecture of the E. coli Chemosensory Array
Source: Biomolecules. 2021 Mar 25;11(4):495. doi: 10.3390/biom11040495 (PMC8064477; doi:10.3390/biom11040495)
Supplement: Supplementary file 1 [file biomolecules-11-00495-s001.zip › supplementary_biomolecules-1120003/supplementary-figure-legends.pdf]

Supplementary Figure legends

Supplementary Figure 1
Images of chemosensory arrays in WM4196 minicells. (A) Membranograms of array patches in our dataset generated from SIRT-like filtered tomograms. Scale bars are not provided because the images are showing data projected on curved surfaces. (B) Oblique 10 nm thick slices through denoised tomograms containing arrays with MCPs aligned parallel to (top left) and perpendicular to (bottom left) the optical axes. Their corresponding power spectra are shown on the right.

Supplementary Figure 2
The hexagonal arrangement of receptors is conserved between the p6 and p2 array architectures. Top view of the p6- and p2-symmetric array models with receptor periplasmic domains coloured according to baseplate partner. Receptors bound to CheA.P5 are shown in red, core CheW in yellow, and flanking CheW in blue.

Supplementary Figure 3
Estimation of the average radius of curvature of the inner membrane of a WM4196 minicell. An XZ projection through the region of a tomogram containing a p2-symmetric array architecture. Local estimates for radii of curvature are indicated on an overlaid ellipse. A circle with curvature equal to the mean curvature of the ellipse is plotted as a dashed line.

Supplementary Figure 4
An image region of an *in vitro* reconstituted chemosensory array with very low curvature taken from [24]. Image regions which appear to display a p2 array architecture are highlighted in dashed rectangles. Scale 50 nm.

Supplementary Figure 5
The effects of applying different symmetries to reconstructions centered at the center of six receptor trimers-of-dimers in both p6 and p2 architectures. Applying symmetry during subtomogram averaging experiments on particles centered at the centeres of receptor trimer-of-dimers will perturb the structure differently depending on which ring structure is present in the data. In the p6 array architecture the $(W/A)_3$ ring is 3-fold symmetric, respecting only C1 and C3 symmetries while the $(W)_6$ ring is 6-fold symmetric, respecting C1, C2, C3 and C6 symmetries. The $(W/W/A)_2$ ring in the p2 array architecture is 2-fold symmetric, respecting only C1 and C2 symmetries. The only symmetry common to all ring structures from both p2 and p6 architectures is C1.
