# Supplementary figures and images for: Alternative Architecture of the E. coli Chemosensory Array
Source: Biomolecules. 2021 Mar 25;11(4):495. doi: 10.3390/biom11040495 (PMC8064477; doi:10.3390/biom11040495)

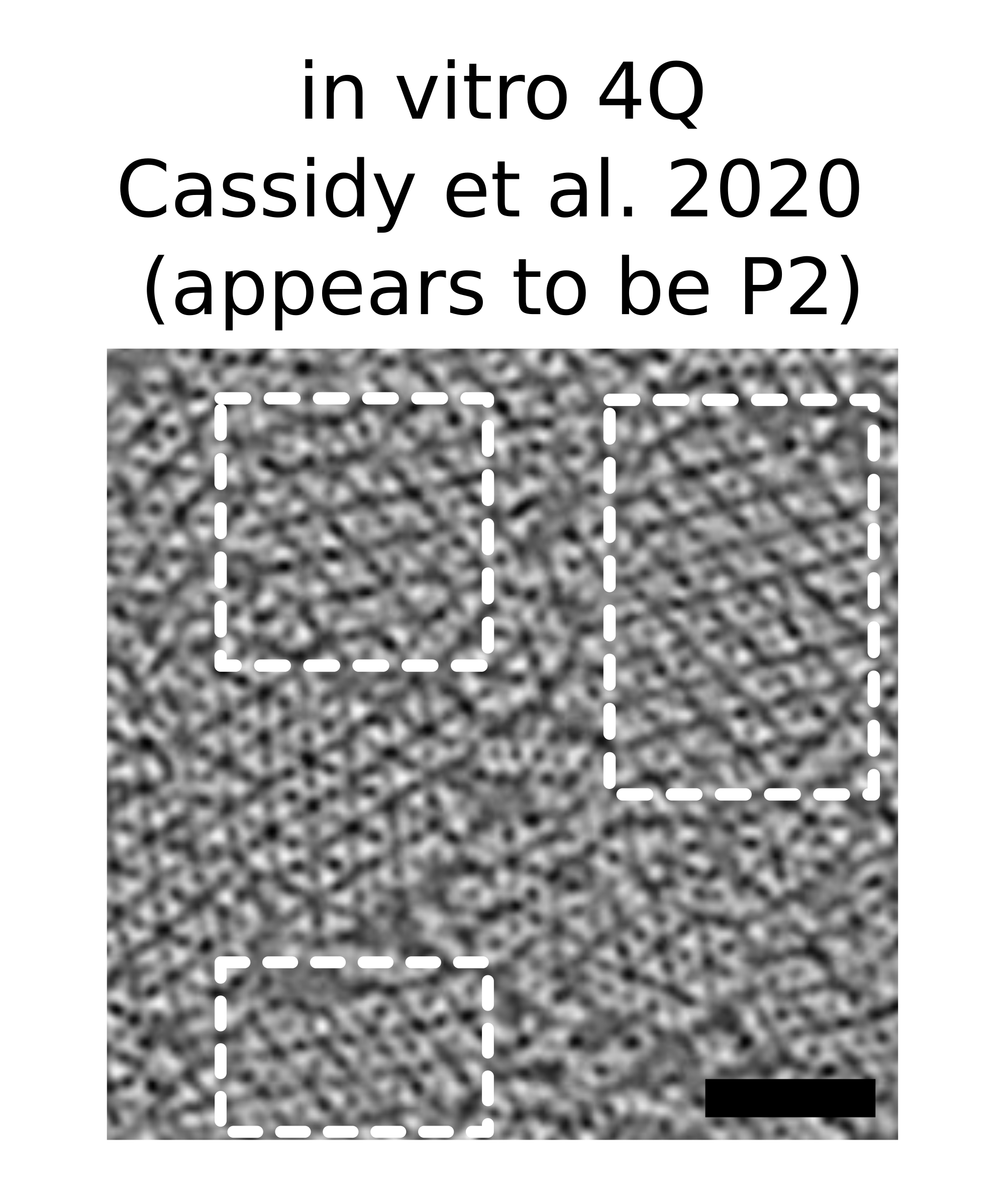

Supplement: Supplementary file 1 [file biomolecules-11-00495-s001.zip › supplementary_biomolecules-1120003/sf4.png]

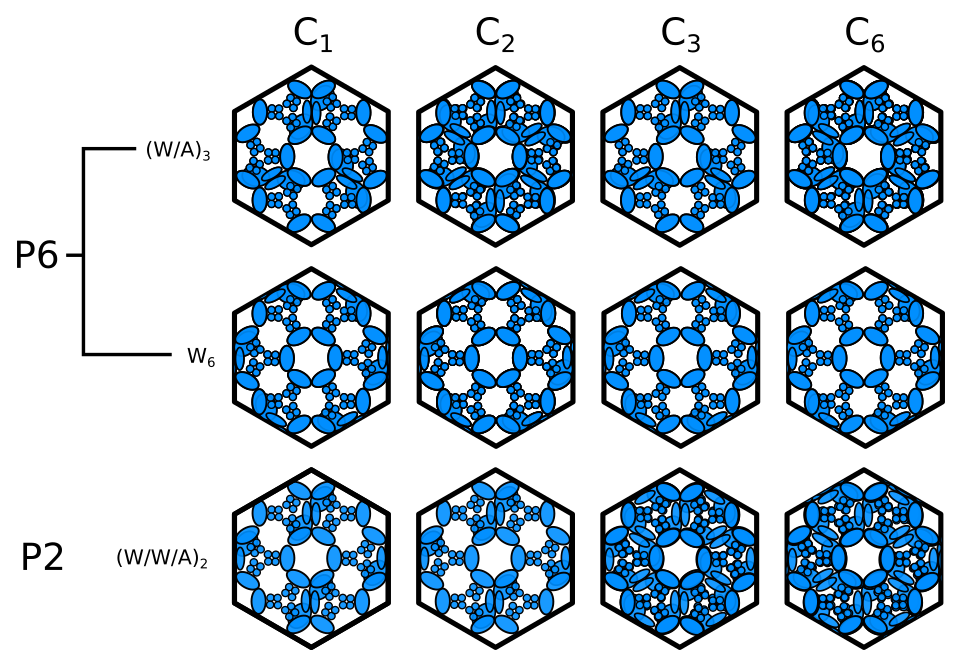

Supplement: Supplementary file 1 [file biomolecules-11-00495-s001.zip › supplementary_biomolecules-1120003/sf5.png]

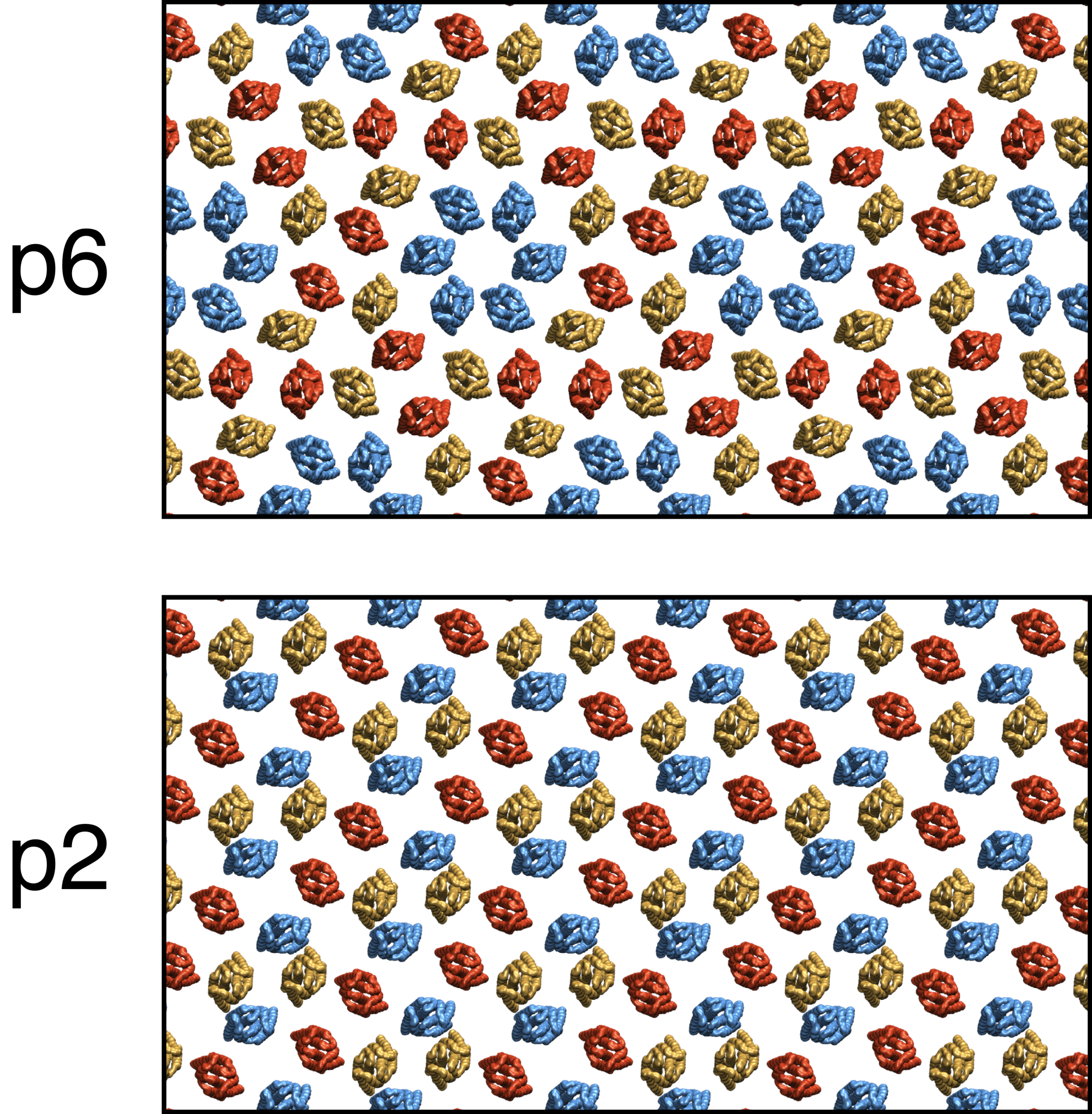

Supplement: Supplementary file 1 [file biomolecules-11-00495-s001.zip › supplementary_biomolecules-1120003/sf2.png]

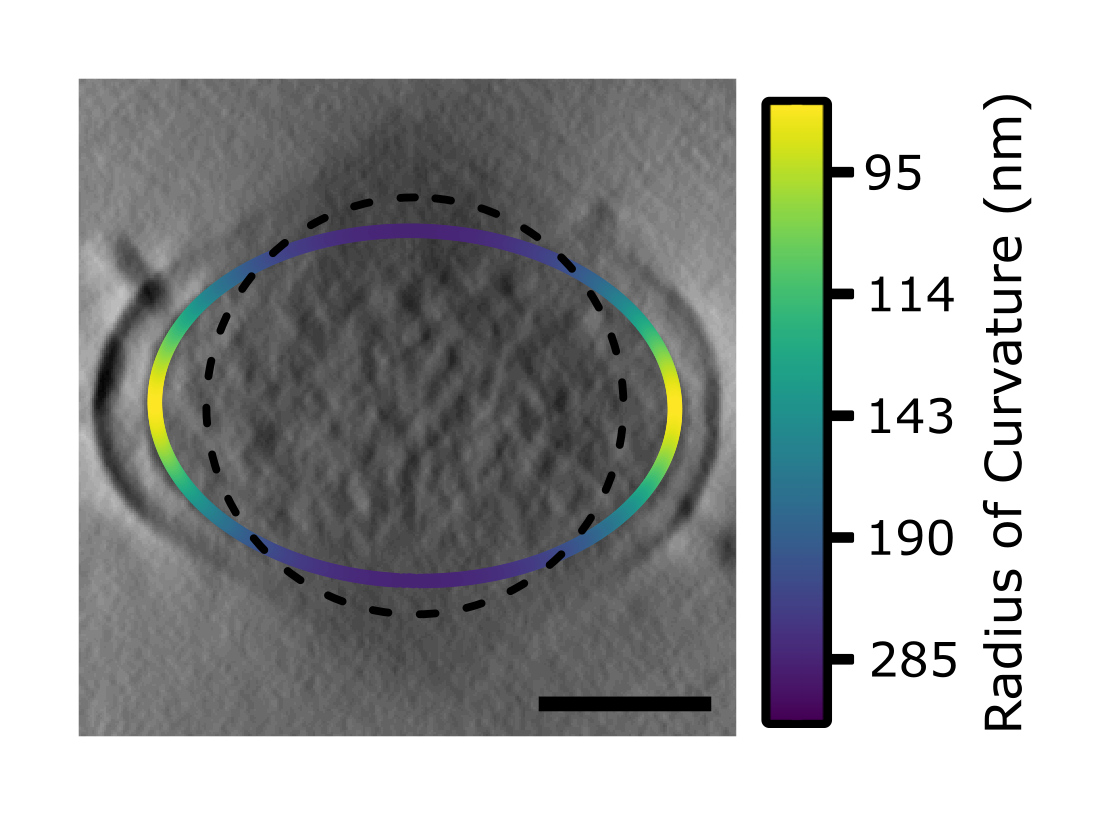

Supplement: Supplementary file 1 [file biomolecules-11-00495-s001.zip › supplementary_biomolecules-1120003/sf3.png]

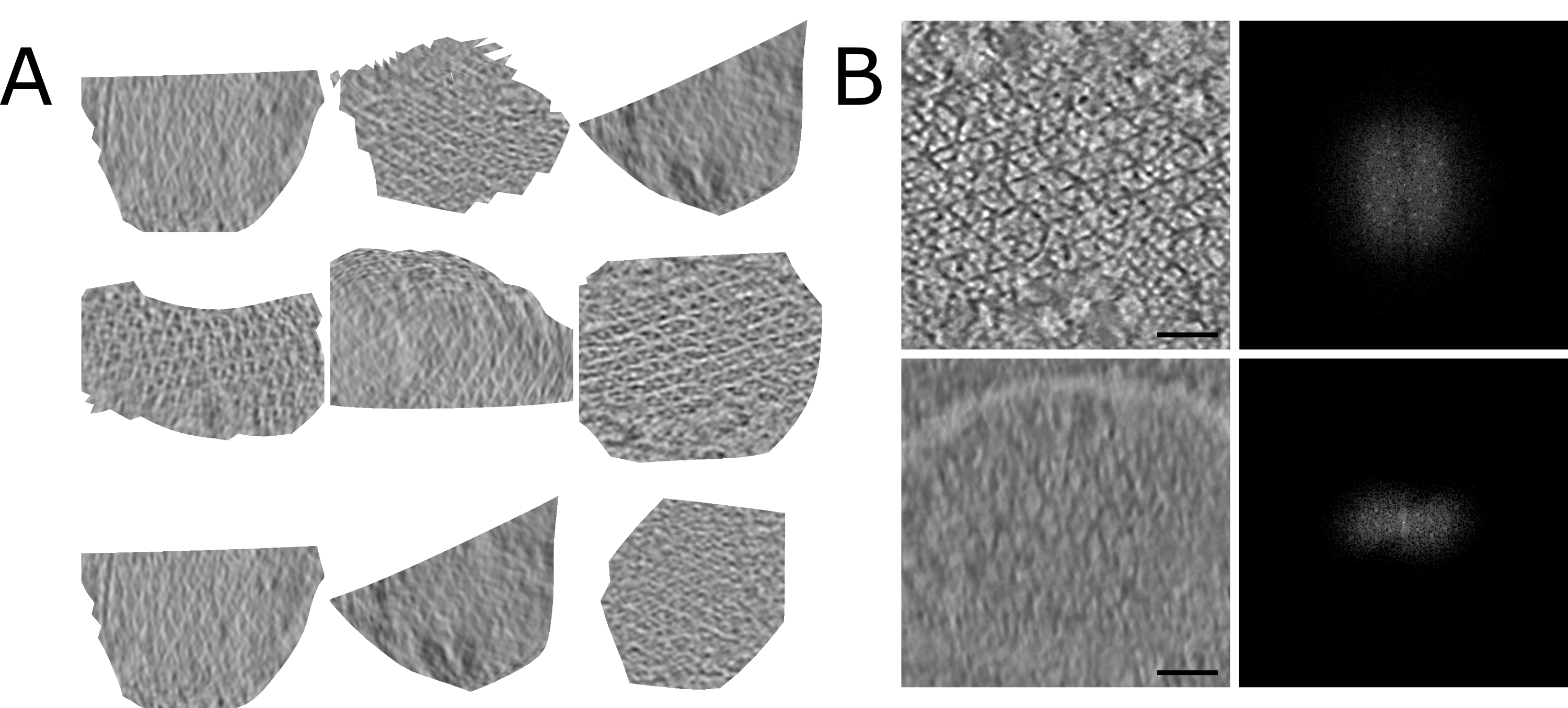

Supplement: Supplementary file 1 [file biomolecules-11-00495-s001.zip › supplementary_biomolecules-1120003/sf1.png]
